# Asthma prevalence based on the Baidu index and China's Health Statistical Yearbook from 2011 to 2020 in China

**DOI:** 10.3389/fpubh.2023.1221852

**Published:** 2023-10-04

**Authors:** Yahui Li, Ping Wang, Xuekun Shao, Fulai Peng, Danyang Lv, Haitao Du, Yi Wang, Xingchen Wang, Fengxia Wu, Cai Chen

**Affiliations:** ^1^First Clinical College, Shandong University of Traditional Chinese Medicine, Jinan, China; ^2^Shandong Academy of Chinese Medicine, Jinan, China; ^3^College of Pharmacy, Shandong University of Traditional Chinese Medicine, Jinan, China; ^4^Shandong Institute of Advanced Technology, Chinese Academy of Sciences, Jinan, China; ^5^School of Basic Medical Sciences, Shandong University, Jinan, China

**Keywords:** asthma, prevalence, Baidu index, gender, age

## Abstract

**Background:**

Due to environmental pollution, changes in lifestyle, and advancements in diagnostic technology, the prevalence of asthma has been increasing over the years. Although China has made early efforts in asthma epidemiology and prevention, there is still a lack of unified and comprehensive epidemiological research within the country. The objective of the study is to determine the nationwide prevalence distribution of asthma using the Baidu Index and China's Health Statistical Yearbook.

**Methods:**

Based on China's Health Statistical Yearbook, we analyzed the gender and age distribution of asthma in China from 2011 to 2020, as well as the length of hospitalization and associated costs. By utilizing the Baidu Index and setting the covering all 31 provinces and autonomous regions in China, we obtained the Baidu Index for the keyword 'asthma'. Heatmaps and growth ratios described the prevalence and growth of asthma in mainland China.

**Results:**

The average expenditure for discharged asthma (standard deviation) patients was ¥5,870 (808). The average length of stay (standard deviation) was 7.9 (0.38) days. During the period of 2011 to 2020, hospitalization expenses for asthma increased while the length of hospital stay decreased. The proportion of discharged patients who were children under the age of 5 were 25.3% (2011), 19.4% (2012), 16% (2013), 17.9% (2014), 13.9% (2015), 11.3% (2016), 10.2% (2017), 9.4% (2018), 8.1% (2019), and 7.2% (2020), respectively. The prevalence of asthma among boys was higher than girls before the age of 14. In contrast, the proportion of women with asthma was larger than men after the age of 14. During the period from 2011 to 2020, the median [The first quartile (Q1)-the third quartile (Q3)] daily asthma Baidu index in Guangdong, Beijing, Jiangsu, Sichuan, and Zhejiang were 419 (279–476), 328 (258–376), 315 (227–365), 272 (166–313), and 312 (233–362) respectively. Coastal regions showed higher levels of attention toward asthma, indicating a higher incidence rate. Since 2014, there has been a rapid increase in the level of attention toward asthma, with the provinces of Qinghai, Sichuan, and Guangdong experiencing the fastest growth.

**Conclusion:**

There are regional variations in the prevalence of asthma among different provinces in China, and the overall prevalence of asthma is increasing.

## 1. Introduction

Bronchial asthma is a diverse chronic airway inflammatory disease involving a wide range of cells, such as eosinophils, mast cells, T lymphocytes, and cell components. The main clinical symptoms typically include repeated attacks during the night and in the morning, or worsening breathing, shortness of breath, and chest tightness (1). Asthmatic patients also have variable airflow restriction and hyperresponsiveness, which can lead to airway remodeling or a series of structural changes in the airways, over the clinical course (2). There are several common types of asthma. (1) Non-allergic asthma are cases of adult asthma that are not related to allergies (3). The phlegm of these patients may contain neutrophils, eosinophils, or only inflammatory cells. (2) Late-onset asthma is asthma that emerges in adulthood, characterized by persistent airway inflammation (4). (3) Allergic asthma, defined by sensitization to environmental allergens, is the most prevalent phenotype of asthma (5). Clinically, asthma manifests as homogeneous symptoms including cough, wheezing, phlegm production, shortness of breath, and varying degrees of respiratory distress.

Generally, asthma is associated with genetic and environmental factors, such as smoking, indoor and outdoor pollution (6), a damp environment (7), exposure to furry pets (8), extensive use of antibiotics in early childhood (9), occupational exposure (10), and obesity (11). Environmental and indoor air pollution problems are becoming increasingly serious, and the prevalence of asthma is rising year by year. According to an age-period-cohort analysis of effects in China, children aged 0–4 years had the highest incidence of asthma, at 1.83%, and the highest morbidity mortality risk between 2015 and 2019 (12). According to the health questionnaire survey by Banta et al. of 61,625 children aged 1–11 years, the prevalence of asthma was 12.9%, with the highest prevalence of 15.5% in children aged 6–11 years (13). A meta-analysis revealed that with a sample size of 212 814 children, an overall prevalence rate was 2.02% (14). Asthma affects ~14% of children worldwide, which makes it the leading chronic respiratory disease in childhood (15).

At the moment, China's secondary hospitals are equipped with lung function instruments and asthma medications, so primary hospitals have the conditions and ability to diagnose and treat asthma. However, China continues to face challenges in spreading and implementing Global Initiative for Asthma (GINA) guidelines in rural areas. According to studies, the prevalence of asthma in China is low, but the case fatality rate is high, and asthma diagnosis and treatment resources in rural areas are relatively scarce, making the situation dire (16). Asthma diagnosis is not difficult in most cases because it is a common disease, but survey results in some areas show that the rate of missed diagnosis in urban and rural areas is 30% and 50%, respectively (17). The reason for this is that current asthma diagnostic standards emphasize pulmonary ventilation function examination as much as possible to avoid under- or over-diagnosis (18). This does not affect the diagnosis of older children, but because lung function testing is not widely available in some regions, diagnosis is typically made only after a period of diagnostic therapy, making early diagnosis difficult. Currently, studies on the epidemiology of asthma in China are mostly limited to a specific region, with small sample sizes, and the epidemiological characteristics of asthma have not been systematically studied and analyzed.

Researchers are currently investigating low-cost public health event monitoring methods based on internet search data. Disease trend forecasting relies heavily on data mining and real-time data monitoring, such as the prediction of real-world respiratory disease outbreaks during the Coronavirus Disease 2019 pandemic through the analysis of lung disease search volume in Google Trends (19). In China, the Baidu Index boasts the largest user base and serves as the primary internet search tool. As of July 2022, Baidu holds a commanding 71.2% share of the Chinese search engine market, far surpassing other competitors like Google. Furthermore, according to the latest annual report from China's Internet Network Information Center, the number of internet users in China has reached 1.032 billion as of the end of 2021, with nearly 300 million users engaging in online healthcare, accounting for 28.9% of the total internet user population. Over the past decade, the number of internet users in China has steadily increased, and online searching has become an essential means of information retrieval for the majority. Intensifying social competition and demanding work schedules have further prompted people's reliance on internet healthcare, leading to the habitual behavior of turning to Baidu for medical inquiries rather than consulting a doctor when feeling unwell. The calculation method of the Baidu Index involves the collection and analysis of users' search behaviors in the Baidu search engine. It quantifies the search volume of specific keywords in a certain geographical area within a given time range and converts it into a relative index value. A higher index value indicates a higher level of popularity for the keyword. In the context of this study, a higher Baidu Index value for asthma reflects a greater public concern and interest in asthma. China's Health Statistics Yearbook is an annual statistical reference book compiled and published by the National Health Commission of China (formerly the Ministry of Health). It serves as a comprehensive tool for collecting and presenting health data and statistical information from various provinces, autonomous regions, municipalities, and important cities across the country. The yearbook covers a wide range of health-related indicators and data in various domains, as well as health policies, regulations, and standards. It integrates statistics from different fields of health, such as population health and disease control, and is updated and released annually to reflect the latest health data and information. The data in the Chinese Health Statistics Yearbook mainly originates from health monitoring, statistical surveys, and information systems nationwide, which ensures the authority and reliability of the sources.

Because of the rise of the Internet and the advancement of Internet technology, it is now possible to investigate the prevalence of asthma. Previous research on snoring in China, the United States, Russia, and India using the Baidu Index and Google Index revealed that snoring varies with the seasons (20). We investigated the prevalence of asthma in China using China's Health Statistics Yearbook and searches about “asthma” using the Baidu index.

## 2. Method

Our study is divided into two segments. The first segment entails an analysis of data from China's Health Statistics Yearbook, specifically delving into the records of 1,733,515 asthma patients who were discharged. Our analysis encompassed various aspects of asthma, including the mortality rate, gender and age distribution, duration of hospitalization, and expenses incurred during hospitalization. The second segment involved an examination of asthma distribution based on the Baidu search index. We scrutinized the heat maps and growth indices of asthma search trends from 2011 to 2020 across 31 provinces, municipalities, and autonomous regions in mainland China. Descriptive analysis was used to describe asthma discharge and asthma search.

The data provided originates from the hospitalization outcomes of patients with asthma, as documented in China's Health Statistics Yearbook from the years 2011 to 2020. In the yearbook, asthma data are year-by-year, and asthma diagnosis criteria come from the International Classification of Diseases (ICD-10). The dataset comprises information pertaining to the number of patients discharged, average medical expenses incurred, average mortality rate, average length of hospital stay, and age and gender distribution of the patients. China's Health Statistics Yearbook stands as a significant annual compendium in the realm of Chinese health and wellbeing, meticulously crafted and released by the National Health Commission of China. This invaluable reference book gathers and harmonizes statistical data and information from various regions across the nation, encapsulating the current state and evolving trends of China's health and healthcare landscape. It sheds light on critical aspects such as the nation's health profile, healthcare services provision, infectious disease outbreaks, and the state of healthcare institutions and professionals (21). Pearson correlation was utilized to assess the correlation between the duration of hospitalization and the associated expenses.

With the rapid development of Internet technology, long-term and continuous data collection based on the Internet has brought opportunities for disease prediction and early warning, especially for COVID-19 (20, 22). According to China's Internet Network Information Center (CNNIC), from 2017 to 2019, the average usage rate of search engines in all types of Internet applications was 82.12%, and the brand penetration rate of the Baidu search engine was 90.9%, showing it has become an important window for Chinese citizens to obtain information. Thus, in this study, asthma data was attained from the Baidu Index from 2011 to 2020 to analyze the distribution of asthma in China. In this study, we obtained the Baidu index of asthma in 31 provinces, municipalities, and autonomous regions in mainland China, and the data were daily data. In order to assess the growth of the asthma search index, we have opted to employ a ratio to depict the pace at which asthma is expanding, as demonstrated by the following formula.

$$\begin{array}{l} \text{Rati}{\text{o}}_{1}=\frac{\text{Baidu index in }2015}{\text{Baidu index in }2011}\\ \text{Rati}{\text{o}}_{2}=\frac{\text{Baidu index in }2020}{\text{Baidu index in }2015} \end{array}$$

## 3. Result

### 3.1. China's health statistical yearbook

**Figure 1:**
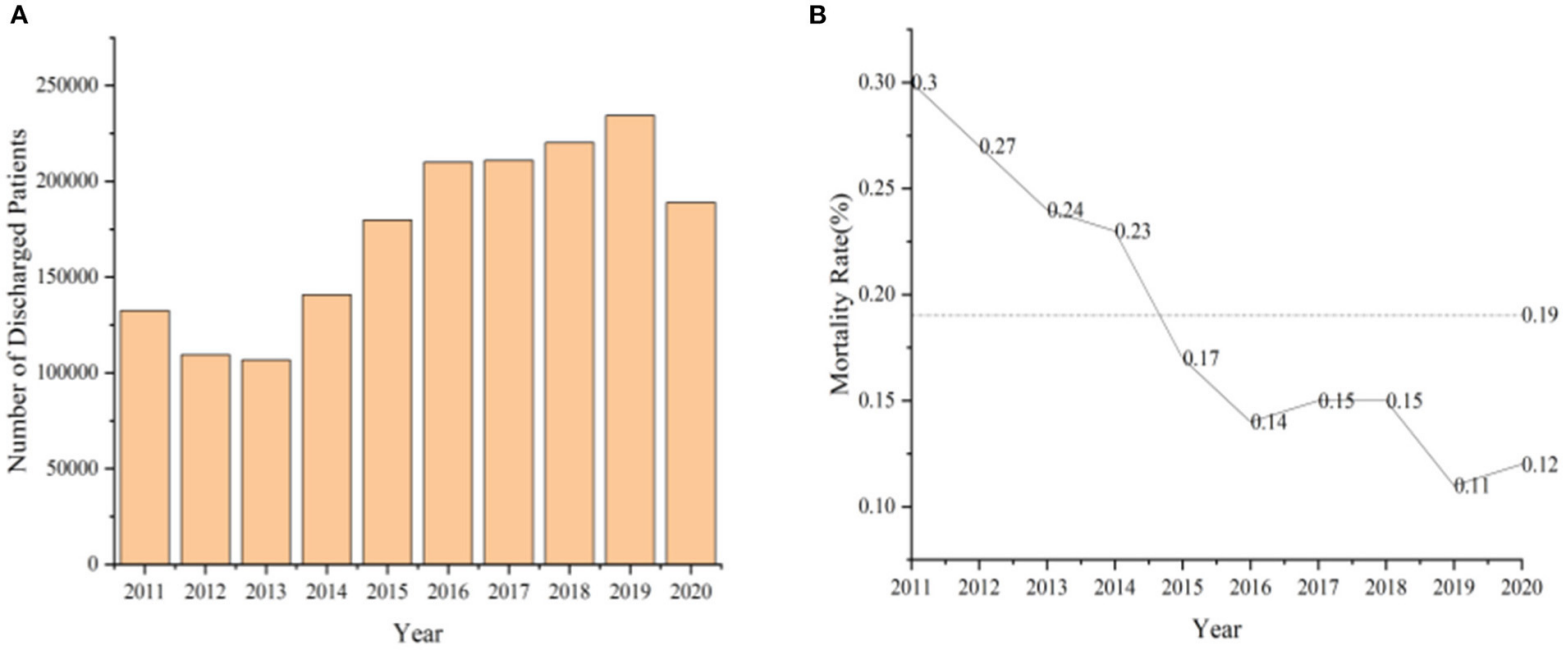
Number of discharged asthma patients **(A)** and mortality rate **(B)** from 2011 to 2020.

In this section, trends in asthma prevalence from 2011 to 2020 according to China's Health Statistics Yearbook are summarized. As shown in Figure 1A, asthma patients had the highest number of hospitalizations in 2019, at 234,343, and the lowest number of hospitalizations were in 2013, at 106,653. The overall asthma mortality rate has been declining, with a death rate of 0.11 in 2019, the lowest in a decade (Figure 1B). As shown in Table 1, the number of discharged patients with asthma in China from 2011 to 2020 were as follows: 132,529 in 2011, 109,475 in 2012, 106,653 in 2013, 140,784 in 2014, 179,844 in 2015, 209,871 in 2016, 210,851 in 2017, 220,177 in 2018, 234,343 in 2019, and 188,988 in 2020. The mortality rates for asthma during the years 2011 to 2020 were 0.3, 0.27, 0.24, 0.23, 0.17, 0.14, 0.15, 0.15, 0.11, and 0.12, respectively. As can be seen from Figure 1B, there is a decreasing trend in mortality due to asthma over this period despite a slight increase observed in 2017, 2018, and 2020.

**Table 1:** Description analysis of China's health statistical yearbook from 2011 to 2020.

**Year**	**number of discharged patients**	**Mortality (%)**	**Average length of stay (day)**	**Average hospitalization cost (¥)**
2011	132,529	0.3	8.4	4,351
2012	109,475	0.27	8.7	4,736
2013	106,653	0.24	7.9	5,445
2014	140,784	0.23	8.12	5,708
2015	179,844	0.17	7.93	6,027
2016	209,871	0.14	7.87	6,246
2017	210,851	0.15	7.83	6,462
2018	220,177	0.15	7.7	6,473
2019	234,343	0.11	7.63	6,790
2020	188,988	0.12	7.4	6,461
X ± Std	173,351 ± 47,369	0.18 ± 0.07	7.9 ± 0.38	5,870 ± 808

**Figure 2:**
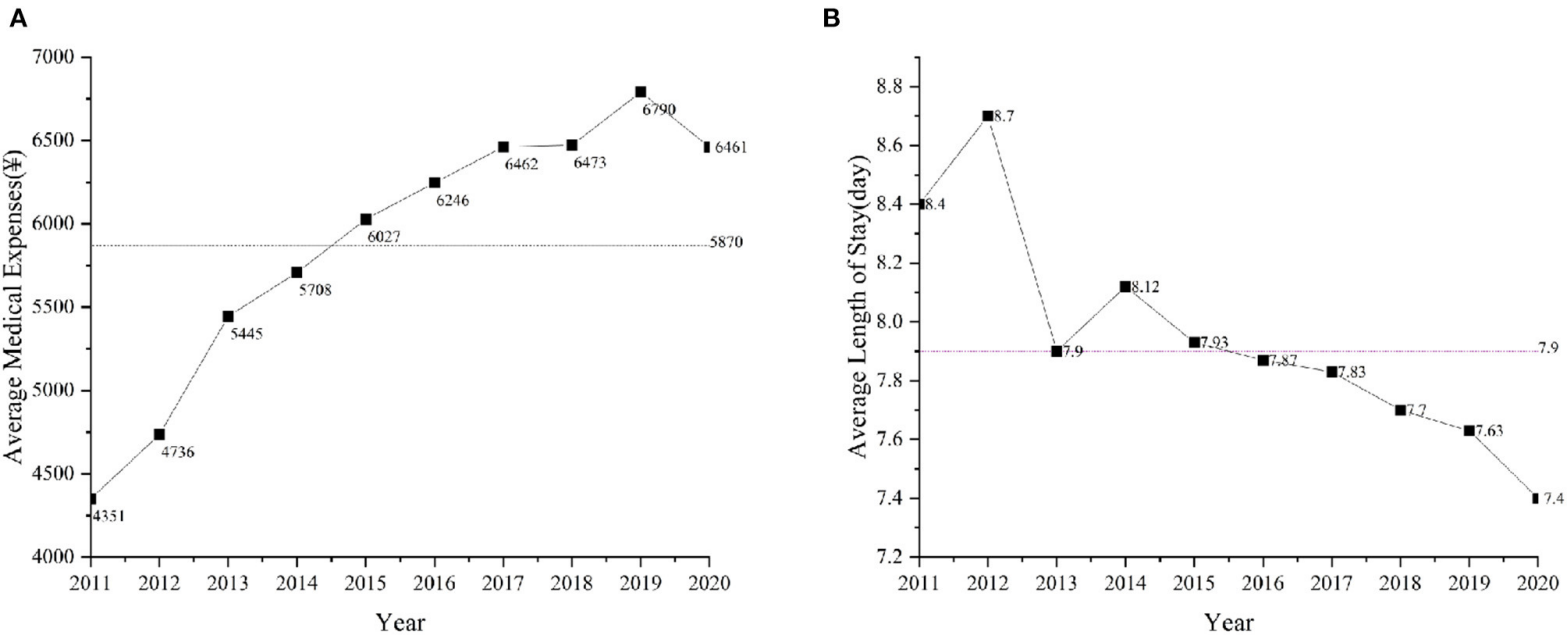
Average medical expense **(A)** and Average length of stay **(B)** from 2011 to 2020.

In contrast to mortality rate, Figure 2A reveals that the average medical expense of discharged patients increased annually. The average expenditure for discharged asthma (standard deviation) patients was ¥5,870 (808). There is a decreasing trend in the average length of stay over the years, and since 2015 hospital stays have been below average. According to Figure 2B, the average length of stay (standard deviation) was 7.9 (0.38) days. The correlation coefficient between the duration of hospitalization and the associated expenses is −0.88 with a *p*-value of 0.0008, suggesting that while the duration of hospitalization has been decreasing, there is an upward trend in the expenses incurred during the stay.

**Figure 3:**
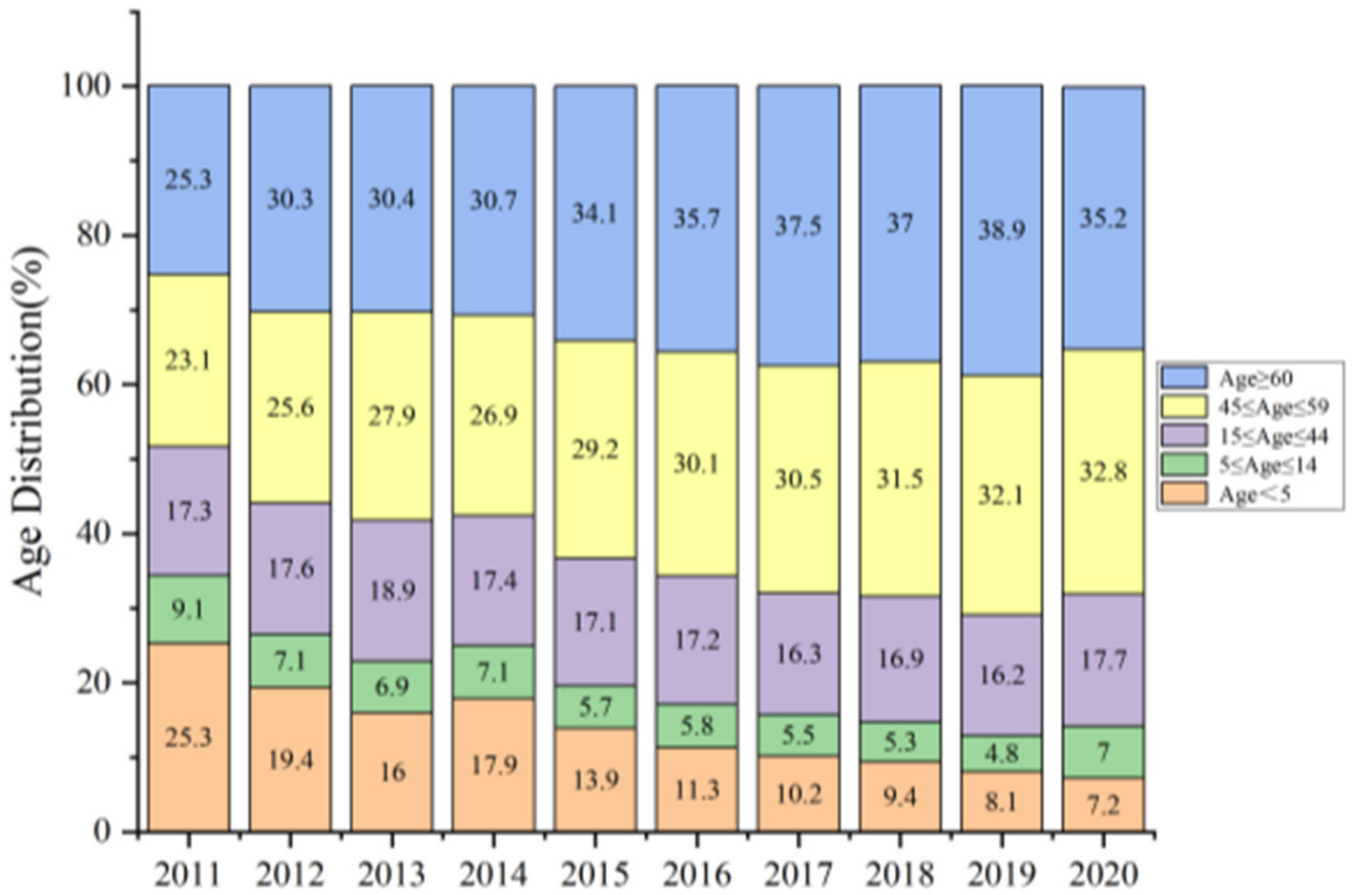
Age distribution from 2011 to 2020.

**Figure 4:**
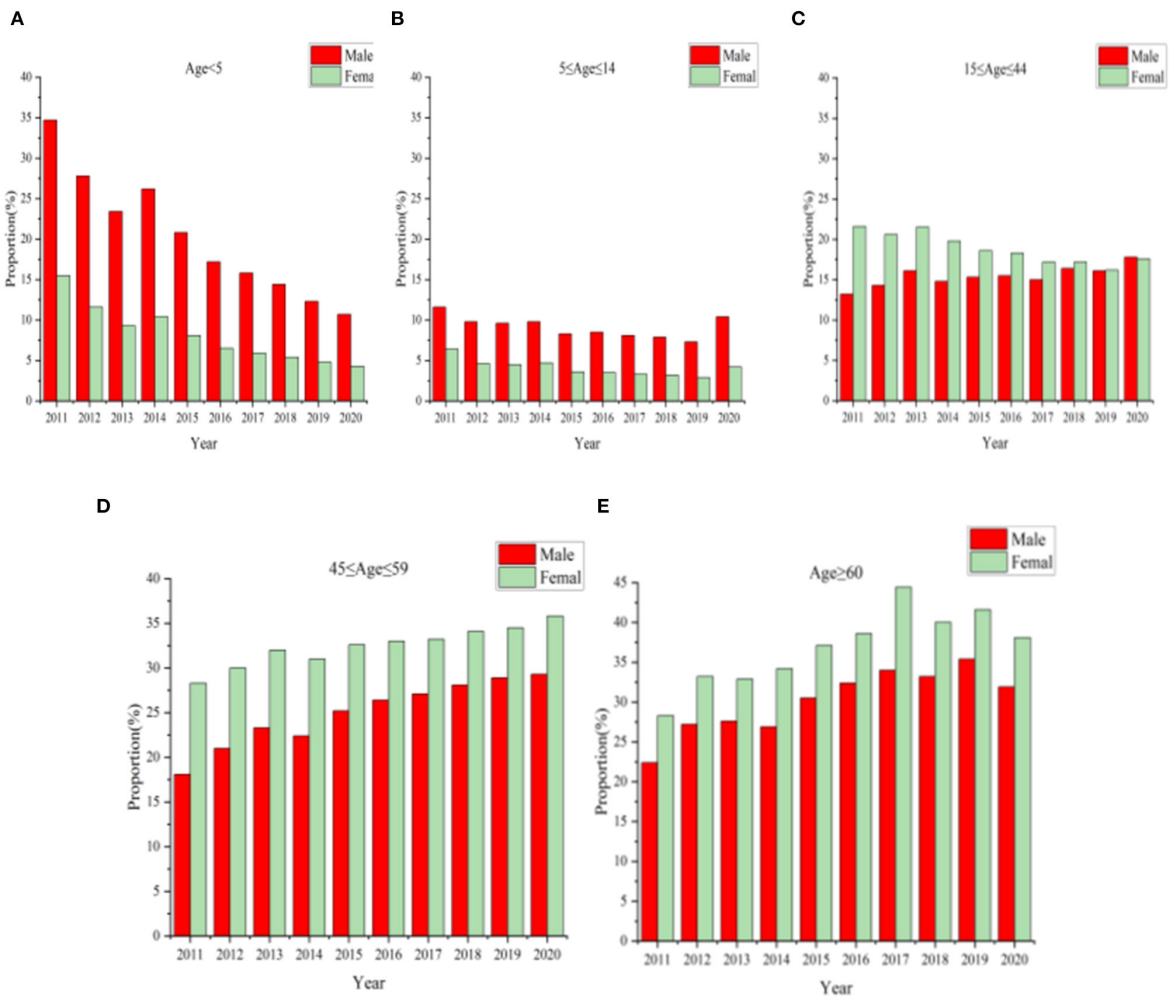
Sex-age distribution from 2011 to 2020.

Figure 3 describes the age distribution about asthma discharge from 2011 to 2020. The proportion of discharged patients who were children under the age of 5 were 25.3%(2011), 19.4%(2012), 16%(2013), 17.9%(2014), 13.9%(2015), 11.3%(2016), 10.2%(2017), 9.4%(2018), 8.1%(2019), and 7.2% (2020), respectively. The maximum proportion of discharged patients of age < 5, 5 ≤ ag e ≤ 14 group, 15 ≤ age ≤ 44 group, 45 ≤ age ≤ 59 group, and age ≥6 0 group in 2011 (25.3%), 2011 (9.1%), 2013 (18.9%), 2020 (32.8%), and 2019 (38.9%), respectively. When asthmatic patients were younger than 14, the hospitalization proportion of boys was greater than that of girls. In contrast, the proportion of women with asthma was larger than that of men after the age of 14 (Figure 4, Table 2). After the age of 45, the rate of discharge for female asthma patients peaked at 44% in 2017, while for male asthma patients, it reached its highest point at 35.8% in 2020.

**Table 2:** Age and sex distribution of discharged patients from 2011 to 2020(%).

	**Age** ≤ **5**		**5**<**Age** ≤ **14**		**15**<**Age** ≤ **44**		**44**<**Age** ≤ **59**		**60** ≤ **Age**	
	**Male**	**Female**	**Male**	**Female**	**Male**	**Female**	**Male**	**Female**	**Male**	**Female**
2011	34.7	15.5	11.6	6.4	13.2	21.6	18.1	28.3	22.4	28.3
2012	27.8	11.6	9.8	4.6	14.3	20.6	21.0	30.0	27.2	33.2
2013	23.4	9.3	9.6	4.5	16.1	21.5	23.3	32.0	27.6	32.9
2014	26.2	10.4	9.8	4.7	14.8	19.8	22.4	31.0	26.9	34.2
2015	20.8	8.1	8.3	3.6	15.3	18.6	25.2	32.6	30.5	37.1
2016	17.2	6.5	8.5	3.5	15.5	18.3	26.4	33.0	32.4	38.6
2017	15.8	5.9	8.1	3.3	15.0	17.2	27.1	33.2	34.0	44.4
2018	14.4	5.4	7.9	3.2	16.4	17.2	28.1	34.1	33.2	40.0
2019	12.3	4.8	7.3	2.9	16.1	16.2	28.9	34.5	35.4	41.6
2020	10.7	4.3	10.4	4.2	17.8	17.6	29.3	35.8	31.9	38.1

### 3.2. Baidu index

**Figure 5:**
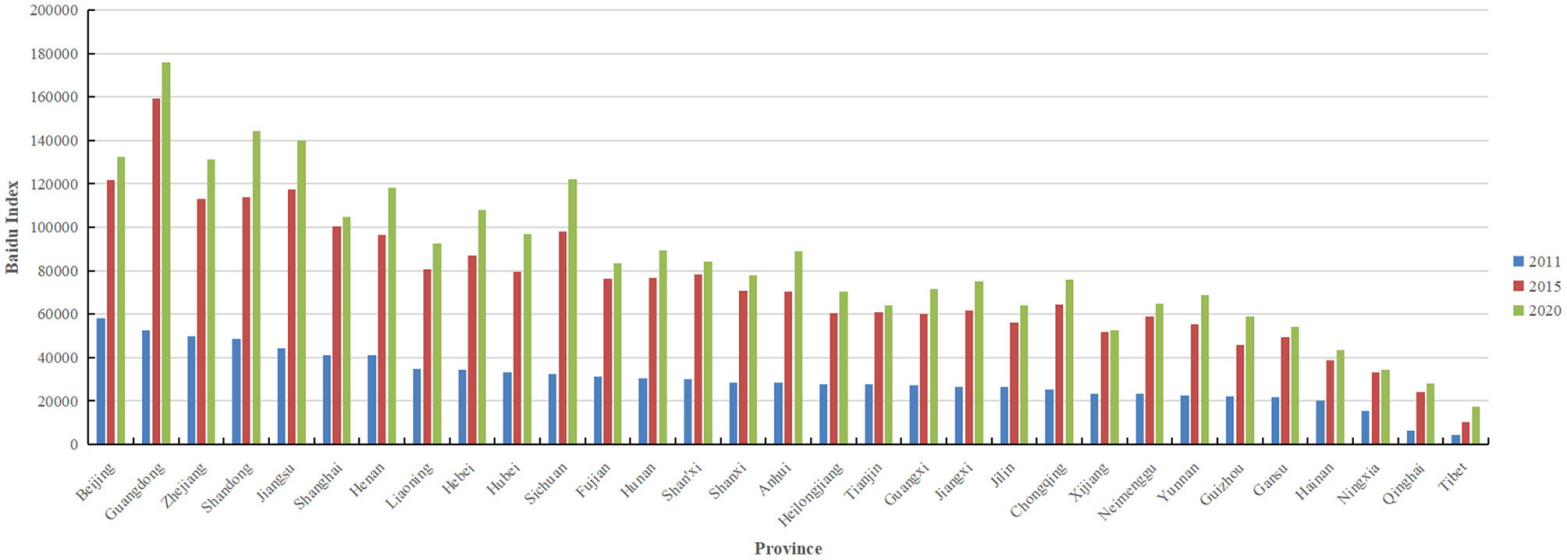
Asthma search index in mainland China in 2011, 2015, and 2020.

**Figure 6:**
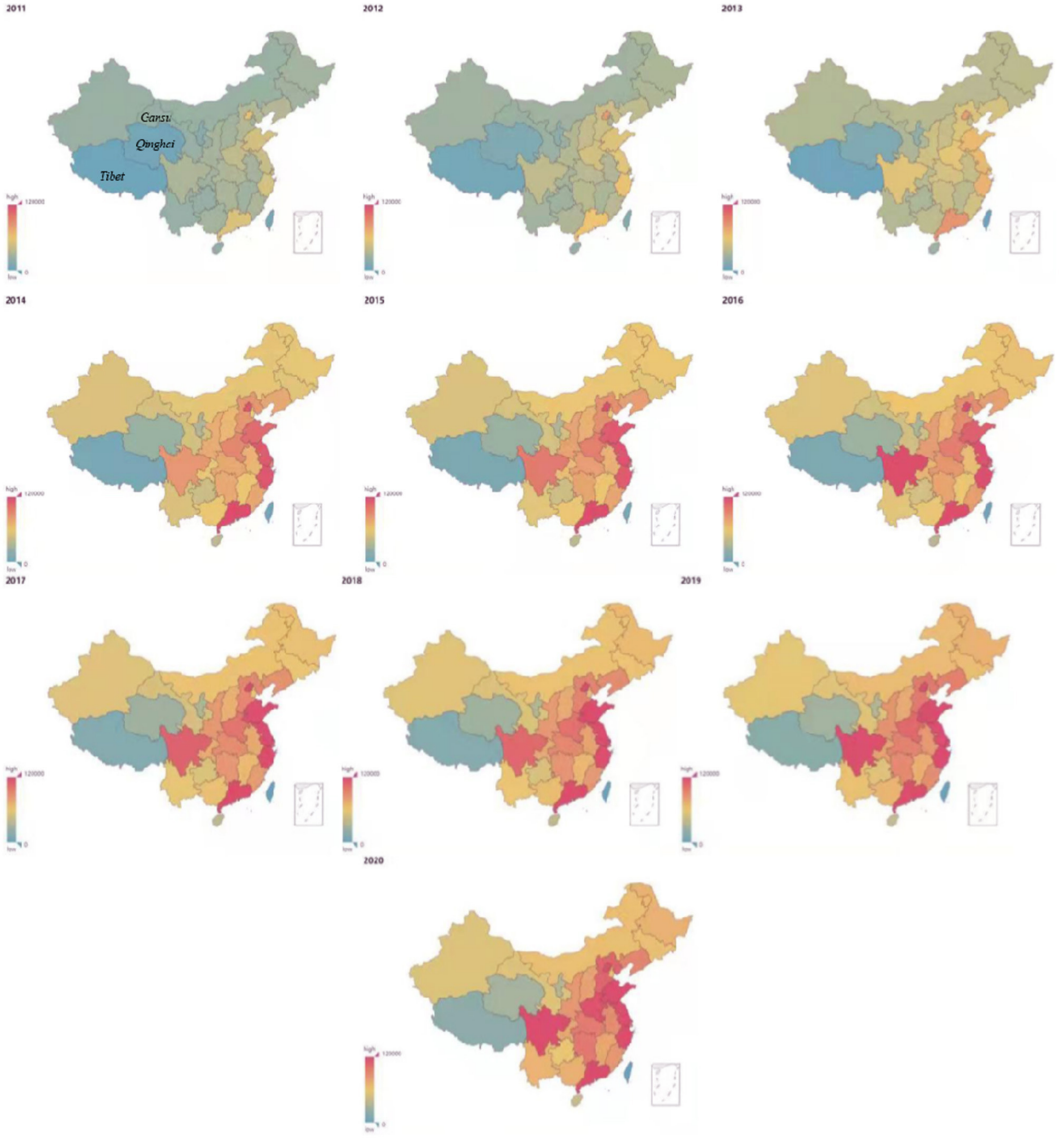
Baidu search index thermodynamic graph from 2011 to 2020.

According to Figure 5, in 2020, the total Baidu Index about asthma in Guangdong, Shandong, Jiangsu, Sichuan, and Beijing was 175,746, 144,366, 139,838, 122,181, and 132,263, respectively. Data from Table 3 show that during the period from 2011 to 2020, the median [The first quartile (Q1)-the third quartile (Q3)] daily asthma Baidu index in Guangdong, Beijing, Jiangsu, Sichuan, and Zhejiang were 419 (279–476), 328 (258–376), 315 (227–365), 272 (166–313), and 312 (233–362) respectively. Throughout this period, the median (Q1–Q3) daily asthma Baidu index in Inner Mongolia, Shandong, Shanghai, Henan, and Liaoning stood at 156 (87–117), 298 (221–345), 262 (179–304), 257 (190–287), and 219 (155–245) respectively. Figure 6 illustrates asthma data from the Baidu Index. From 2011 to 2013, the population paid little attention to asthma, particularly in Tibet, Qinghai, and Gansu provinces. However, the number of people searching for asthma has increased rapidly since 2014, indicating that people are becoming more aware of the disease, particularly in Guangdong, Jiangsu, Zhejiang, Shandong, Beijing, and Sichuan. Overall, awareness of asthma has remained high since 2014, and economically developed provinces in coastal areas are more aware than those in inland areas. Among inland provinces, Sichuan province had the highest asthma search index since 2011.

**Table 3:** Descriptive results of the asthma Baidu index from 2011 to 2020.

**No**.	**Province**	**Q3**	**Median**	**Q1**	**Mean**	**Std**	**No**.	**Province**	**Q3**	**Median**	**Q1**	**Mean**	**Std**	**No**.	**Province**	**Q3**	**Median**	**Q1**	**Mean**	**Std**
1	Guangdong	476	419	279	375	149	11	Hebei	261	236	175	214	73	21	Chongqing	207	179	99	161	59
2	Beijing	376	328	258	312	97	12	Ningxia	117	67	60	78	38	22	Yunnan	174	152	87	136	49
3	Jiangsu	365	315	227	290	108	13	Shanxi	208	185	126	169	58	23	Jilin	174	155	104	142	44
4	Sichuan	313	272	166	242	98	14	Shan'xi	230	210	131	186	64	24	Guangxi	184	165	112	150	47
5	Zhejiang	362	312	233	291	100	15	Hubei	248	221	154	199	69	25	Gansu	157	133	74	119	45
6	Inner Mongolia	177	156	87	142	57	16	Anhui	218	189	121	173	61	26	Tianjin	177	161	102	147	43
7	Shandong	345	298	221	277	98	17	Hunan	235	210	138	188	65	27	Guizhou	154	131	76	120	44
8	Shanghai	304	262	179	243	87	18	Fujian	229	210	155	187	61	28	Xinjiang	160	143	82	125	43
9	Henan	287	257	190	235	80	19	Jiangxi	192	168	100	152	52	29	Hainan	131	82	64	95	38
10	Liaoning	245	219	155	198	65	20	Heilongjiang	186	165	105	151	48	30	Qinghai	66	61	57	57	35
														31	Tibet	57	0	0	30	32

Q3, the third quartile; Q1, the first quartile; Std, standard deviation.

**Figure 7:**
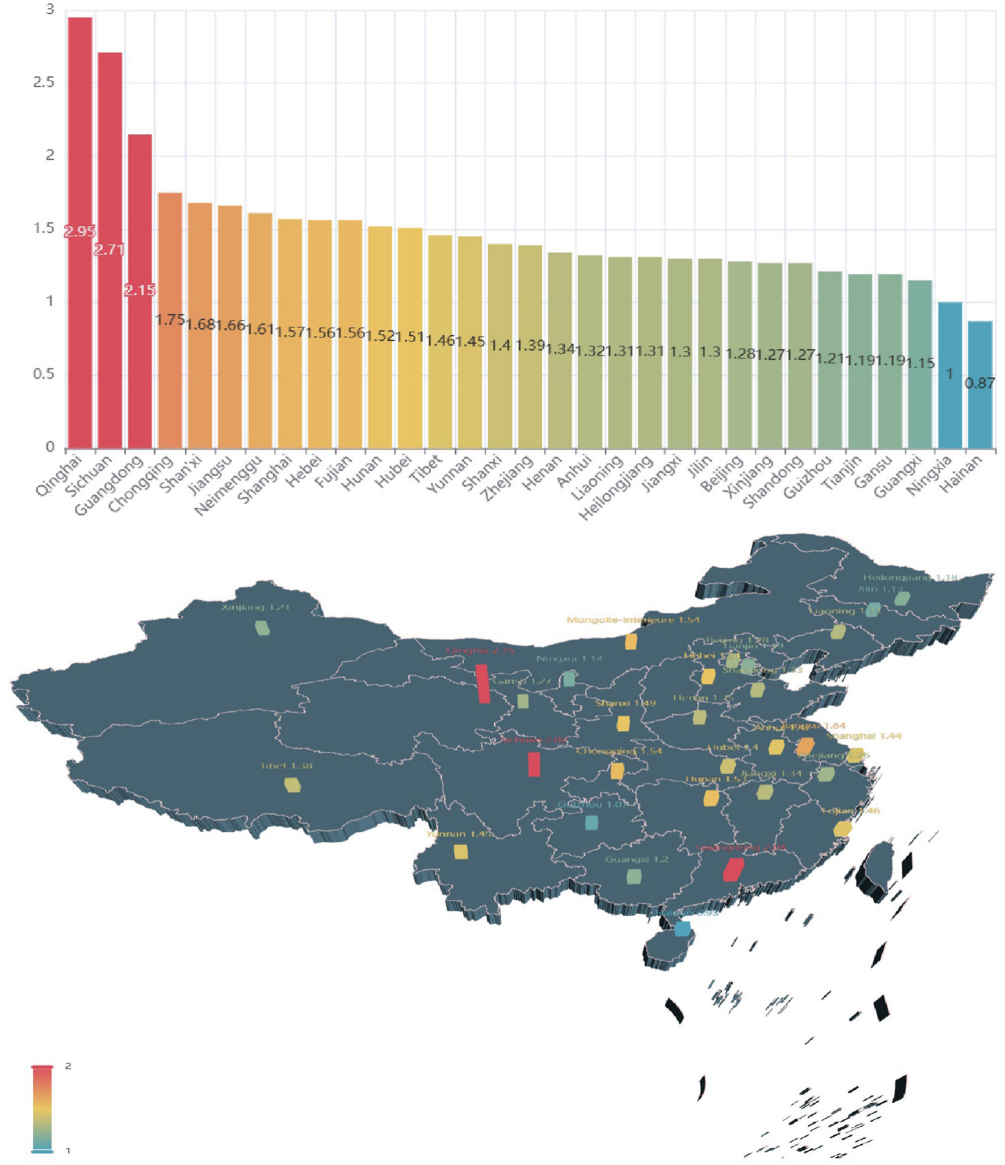
Growth of the search index about asthma in each province between 2011 and 2015.

**Figure 8:**
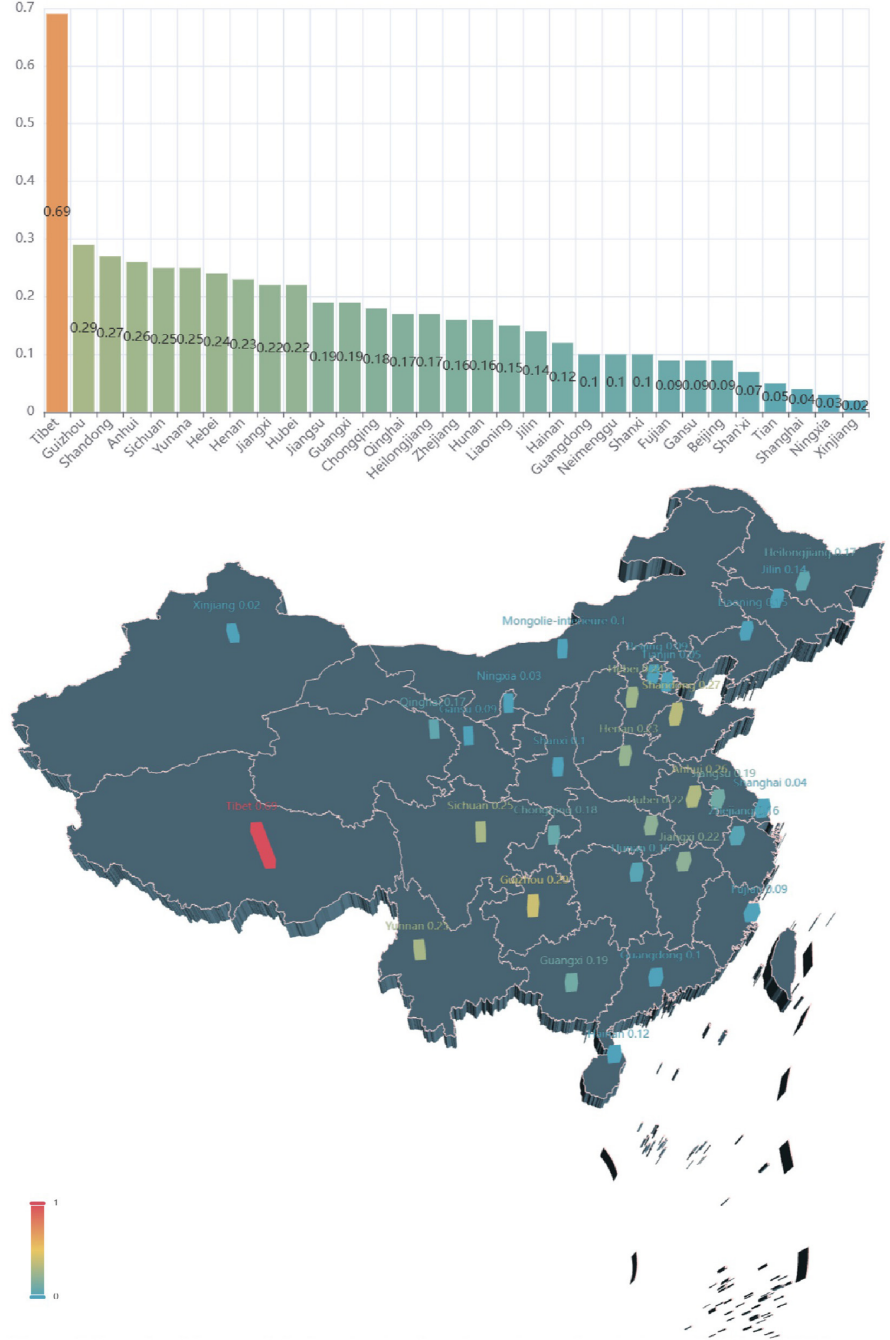
Growth of the search index about asthma in each province between 2015 and 2020.

Figure 7 illustrates the growth of the search index about asthma in each province between 2011 and 2015. All provinces (except Hainan) have more than doubled their attention to asthma since 2011. The top three provinces are Qinghai, Sichuan, and Guangdong, which experienced the fastest growth with rates of 2.95, 2.71, and 2.15 times higher, respectively. Guangxi, Ningxia, and Hainan have lower growth rates than the other provinces, at 1.15, 1 and 0.87 times, respectively. The growth rates of asthma concern in Guangdong, Fujian, Zhejiang, Jiangsu, Shandong, Tianjin, and Liaoning coastal areas were 2.15, 1.56, 1.39, 1.66, 1.27, 1.19, and 1.31, respectively. Figure 8 shows the growth of the search index about asthma in each province between 2015 and 2020. One interesting phenomenon is that growth of the search index about asthma in Tibet is larger than the other provinces, which has risen to 0.69. Shandong, Anhui, and Sichuan province still maintain a fast growth rate compared to that in 2015, with growth rates of 0.27 (Shandong), 0.26 (Anhui), and 0.25 (Sichuan).

## 4. Discussion

Asthma is a long-term respiratory disease that affects both children and adults. Asthma prevalence ranged from 1.1% to 11% in mainland China (23, 24). Of course, it takes time and effort for researchers to thoroughly investigate the prevalence of asthma in each region, and it necessitates the collaboration of multiple teams. With the advent of the Internet, it is now possible to investigate the prevalence of diseases in various regions. China's Health Statistical Yearbook was employed to analyze the discharge of asthma patients from 2011 to 2020, and the Baidu index was utilized to analyze the distribution of asthma in mainland China.

Through analyzing the Baidu search index for asthma, our research has discovered an increase in public interest regarding asthma, suggesting an escalating prevalence of the condition. Indeed, asthma prevalence in our country has shown a rapid upward trend. The results of the third national survey on childhood asthma in China revealed a 3.02% prevalence among urban children, indicating a 52.8% increase since 2001 (25). Meta-analysis demonstrates an overall rising trend in asthma prevalence among Chinese children aged 0 to 14, with higher rates in boys than girls (26). Currently, asthma control in our country still lags behind the treatment levels recommended by GINA. Taking Beijing as an example, only 34.9% of asthma patients have achieved control (27). A study published in The Lancet in 2019 indicated an estimated 45.7 million adult asthma patients in China, with nearly 70% remaining undiagnosed and 95% receiving inadequate standardized treatment (23). The results of a nationwide multicenter survey conducted from 2015 to 2016 showed an overall asthma control rate of 28.5% in urban areas in 2016 (28). Based on questionnaire-based surveys, only 28.7% and 45.0% of patients achieved control and partial control, respectively. Only 21.8% used a peak flow meter (PFM), with 6.6% of patients using it daily (29). Insufficient utilization of peak flow meters indicates a lack of actual implementation for assessing asthma severity and adjusting medication standards based on peak expiratory flow rates. This suggests a low diagnosis rate and high rate of missed asthma cases in China.

**Figure 9:**
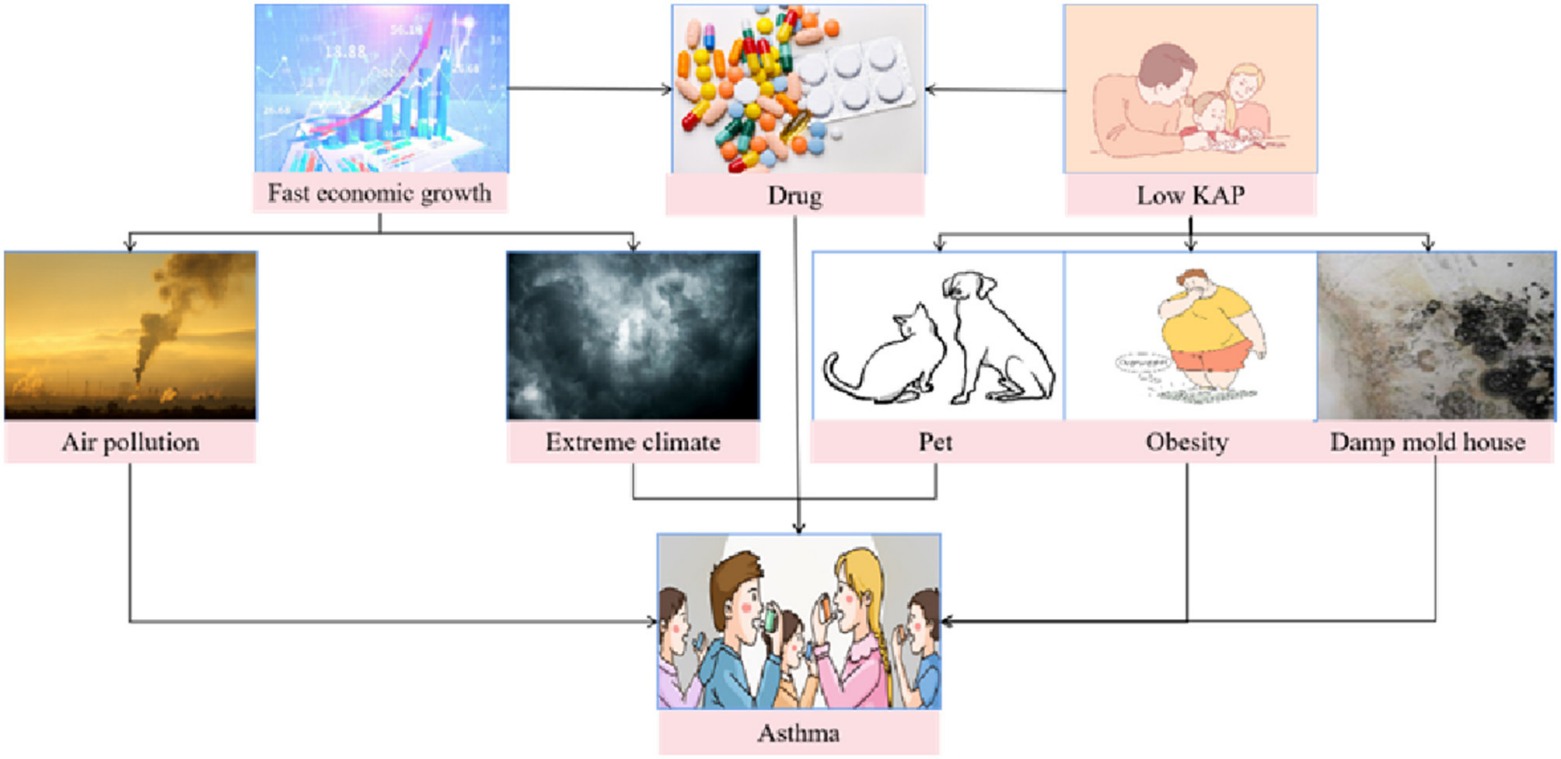
Potential risk factors for asthma.

In this study, we found a gradual increase in asthma hospital discharges, a decrease in mortality, and an increase in hospitalization costs from 2011 to 2020. Foremost, China's remarkable economic growth from 2011 to 2020 has resulted in an escalation of disposable income among its people. We think it could be related to economic growth, advances in drugs and medical technology, low score about knowledge, attitudes and practices (Figure 9). With a growing emphasis on improved healthcare outcomes and services, there is a potential correlation with an upward trajectory in hospital admissions and subsequent discharges. Another reason might be an increase in diagnosis or changes in diagnostic criteria. The advancements in medical technology and a better understanding of asthma may potentially contribute to the increase of diagnosis of asthma as well as allowing a broader spectrum of options for hospitalization as a means of treatment. On the other hand, it could be related to Chinese parents' low knowledge, attitudes, and practices (KAP) scores to asthma. According to Zhao et al. (30), many children in China's capital region have poor asthma control and low parental KAP scores due to factors such as inadequate parental understanding of asthma, exposure to hazardous environments, poor medication adherence, and insufficient regular medical visits (31). Third, even though people have been aware of the harm that air pollution causes to the respiratory system and have made numerous efforts to reduce it, air pollution can still cause asthma in some areas. The idea that outdoor air pollution causes pre-existing asthma to worsen has been supported by decades of accumulating evidence, with some studies suggesting that outdoor air pollution can also cause new cases of asthma (32). Extreme weather events are becoming more common as a result of global climate change, which may also be a factor. According to recent research, extreme temperatures are a significant risk factor for asthma attacks. In extreme heat and cold, the pooled relative risk of asthma attacks was 1.07 (95%CI:1.03–1.12) and 1.20 (95%CI:1.03–1.12), respectively (33). This study also discovered an increase in hospital discharges among people over the age of 60 and an increase in hospitalization costs. We hypothesized that, due to their weakened immunity, older people may be more vulnerable to the influence of the external environment. Furthermore, the basic health condition of some older individuals is not optimistic. Older individuals often have other chronic diseases, such as cardiovascular diseases or other respiratory system ailments. These conditions may interact with asthma, resulting in more complex and persistent asthma attacks, requiring a longer duration for comprehensive treatment and rehabilitation.

Our study found when asthmatic patients were younger than 14, the proportion of boys was greater than that of girls, which indicates that when under the age of 14, boys have a higher incidence of asthma, while after the age of 14, girls have a higher prevalence. This aligns with the findings of previous studies published by other researchers (34–36). Asthma prevalence rises in women compared to men around puberty, and hormonal changes during menstruation, pregnancy, and menopause are linked to changes in asthma symptoms. One possible explanation is that boys' airway synapses develop slowly, which indicates that their airway growth lags behind lung parenchyma growth, resulting in narrower airways than girls (37). Using genetic deletions of estrogen receptors or androgen receptors in animals, researchers discovered that estrogen signaling promotes and androgen signaling inhibits allergen-mediated type 2 airway inflammation (38).

In this research, we discovered that people in China's eastern coastal areas were more concerned about asthma than those in the country's interior. The eastern coastal areas of China have a high level of economic development, a high level of air pollution, and a high level of urbanization, and the majority of them follow modern Western lifestyles. Adoption of Western lifestyles is associated with changes in the environment, behavior, and diet, as seen by increasing indoor time, antibiotic usage, an obesity epidemic, and a decline in physical activity (39). This lifestyle could contribute to the high prevalence of asthma. Asthma prevalence is rising worldwide, particularly in urban areas with heavy industrialization, such as eastern coastal areas of China. Asthma and exposure to air pollution have been linked in cross-sectional and longitudinal studies (23). China is one of the most polluted countries as a result of its rapid industrialization and urbanization. The Chinese government's policy interventions in recent years have led to decreased levels of air pollutants (40). However, it is still significantly higher than in most developed countries. Over the last few decades, the prevalence and burden of asthma in adults (and children) has increased in China (23). A study evaluating the relationship between particulate matter and lung function in children with asthma in Shanghai discovered a strong negative association. The effect of particular matter (PM) is largest in the single pollutant model; that is, when the concentration of PM increases by 1 g/m, forced vital capacity (FVC) and FEV0 fall by 0.91% and 1.05%, respectively (41). According to a study conducted in Fuzhou, China, SO_2_ has the greatest impact on the emergency department of asthma (RR = 1.495, 95%CI:1.241–1.800) and has a greater impact on the outpatient service of children aged 0–4 years than children aged 5–13 years (42).

In this research, we extensively utilized internet search tools to investigate the prevalence of asthma and discovered variations in its occurrence across different regions. Additionally, we made full use of publicly available data from China's Health Yearbook to retrospectively examine the trends in asthma prevalence from 2011 to 2020 among hospitalized patients in China. Undeniably, our research does have certain limitations. The data on asthma that we obtained originated from internet search indices, which, while providing an objective reflection of regional asthma prevalence, still do not represent actual real-world asthma data.

## 5. Conclusion

Asthma is the most common chronic disease in children and one of the leading causes of hospitalization. As a result of environmental pollution, lifestyle changes, and diagnostic advancements, the prevalence of asthma in children worldwide is increasing annually. According to the Baidu Index and China's Health Statistical Yearbook, we examined the prevalence of asthma in China from 2011 to 2020. Before the age of 14, males had a lower prevalence of asthma than females, and coastal areas had a higher concern for asthma. There are regional variations in the prevalence of asthma among different provinces in China.

## Data availability statement

The raw data supporting the conclusions of this article will be made available by the authors, without undue reservation.

## Author contributions

Guarantor of integrity of entire study: XW, FW, and CC. Study concept: CC, YL, PW, and XS. Study design: YL and FP. Literature research: YL, HD, and YW. Data acquisition: DL, CC, YL, and PW. Data analysis: YL and XS. Manuscript preparation: YL. Manuscript editing: PW, XW, FW, CC, and YL. Manuscript revision/review: YL, XW, FW, and CC. All authors have participated in this study and consent to publish this article.
